# Supplementary material for: Choice selective inhibition drives stability and competition in decision circuits
Source: Nat Commun. 2023 Jan 10;14:147. doi: 10.1038/s41467-023-35822-8 (PMC9832138; doi:10.1038/s41467-023-35822-8)
Supplement: Supplementary file 1 — Supplementary Information [file 41467_2023_35822_MOESM1_ESM.pdf]

# Supplementary Information for: Choice selective inhibition drives stability and competition in decision circuits

James P. Roach[1,2], Anne K. Churchland[2], and Tatiana A. Engel[1]

[1]Cold Spring Harbor Laboratory, Cold Spring Harbor, NY

[2]Department of Neurobiology, David Geffen School of Medicine, University of California Los Angeles, Los Angeles, CA

Corresponding author e-mail: engel@cshl.edu

December 12, 2022

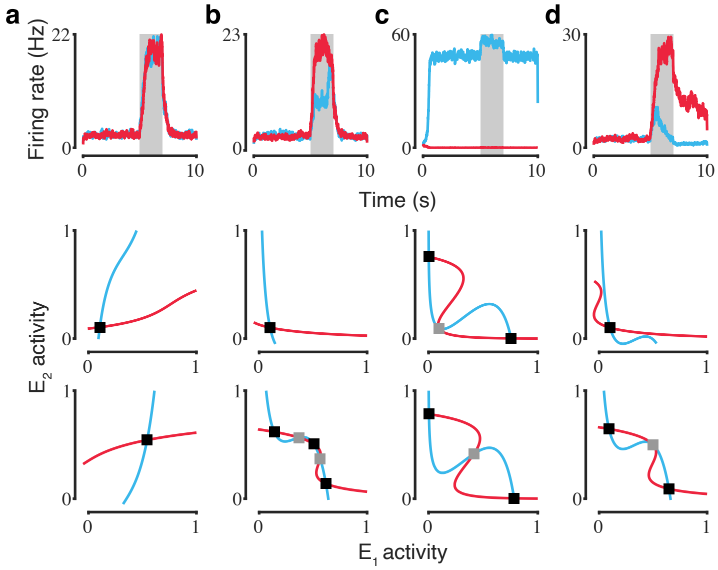


**Supplementary Figure 1. Examples of circuits lacking fixed points necessary for decision making or working memory.** Connection specificity between choice selective populations determines which fixed points are present in the circuit. Example activity traces for choice selective excitatory populations (upper row, gray area indicates stimulation period with 0 stimulus strength) and the phase plane for unstimulated (middle row) and stimulated circuits (lower row, 0 stimulus strength) are shown. Black and grey squares indicate stable attractors and saddle points, respectively. (**a**) A circuit which lacks choice and working memory attractors, as well as a symmetrical saddle point when stimulated. $\gamma_{\rm EE} = 0.175$, $\gamma_{\rm EI} = 0$, $\gamma_{\rm IE} = 0$. (**b**) A circuit which lacks working memory attractors and the symmetrical saddle point. $\gamma_{\rm EE} = 0.175$, $\gamma_{\rm EI} = -0.675$, $\gamma_{\rm IE} = 0.675$. (**c**) A circuit which lacks the symmetrical low activity attractor. $\gamma_{\rm EE} = 0.475$, $\gamma_{\rm EI} = 0$, $\gamma_{\rm IE} = 0$. (**d**) A circuit which lacks working memory attractors. $\gamma_{\rm EE} = 0.35$, $\gamma_{\rm EI} = 0.5$, $\gamma_{\rm IE} = 0.45$.

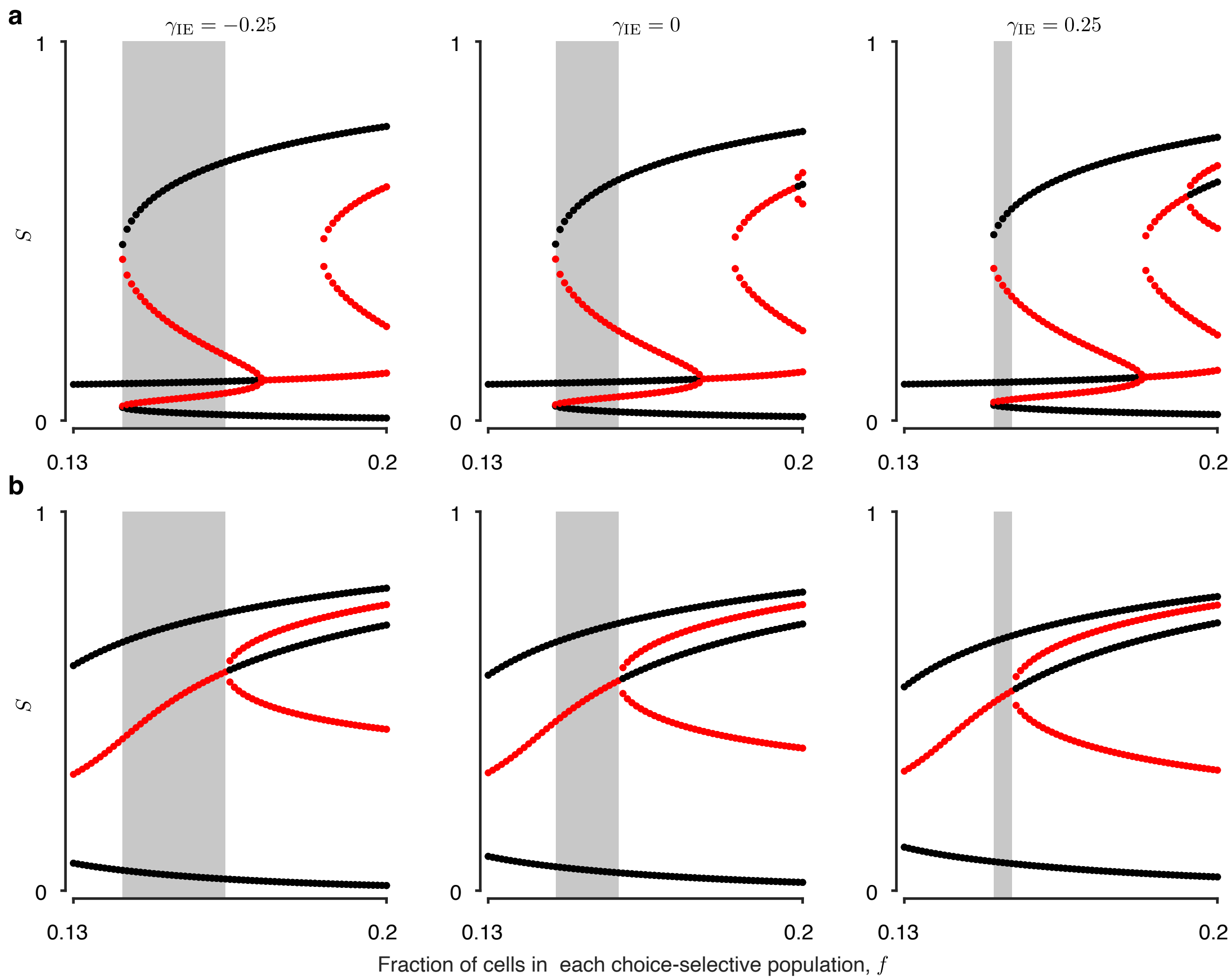


**Supplementary Figure 2. The size of choice-selective populations controls the circuit's ability to support decision making and working memory.** Bifurcation diagrams for (**a**) unstimulated and (**b**) stimulated (stimulus strength equal to 0) circuits when varying the fraction $f$ of selective neurons. The size of selective populations determines which fixed points are present. Contraspecific circuits (left column) tolerate a wider range of selective population sizes than nonspecific (center) and ipsispecific (right) circuits. Grey shading indicates the selective population sizes supporting decision-making as indicated by the presence of the eight fixed points.

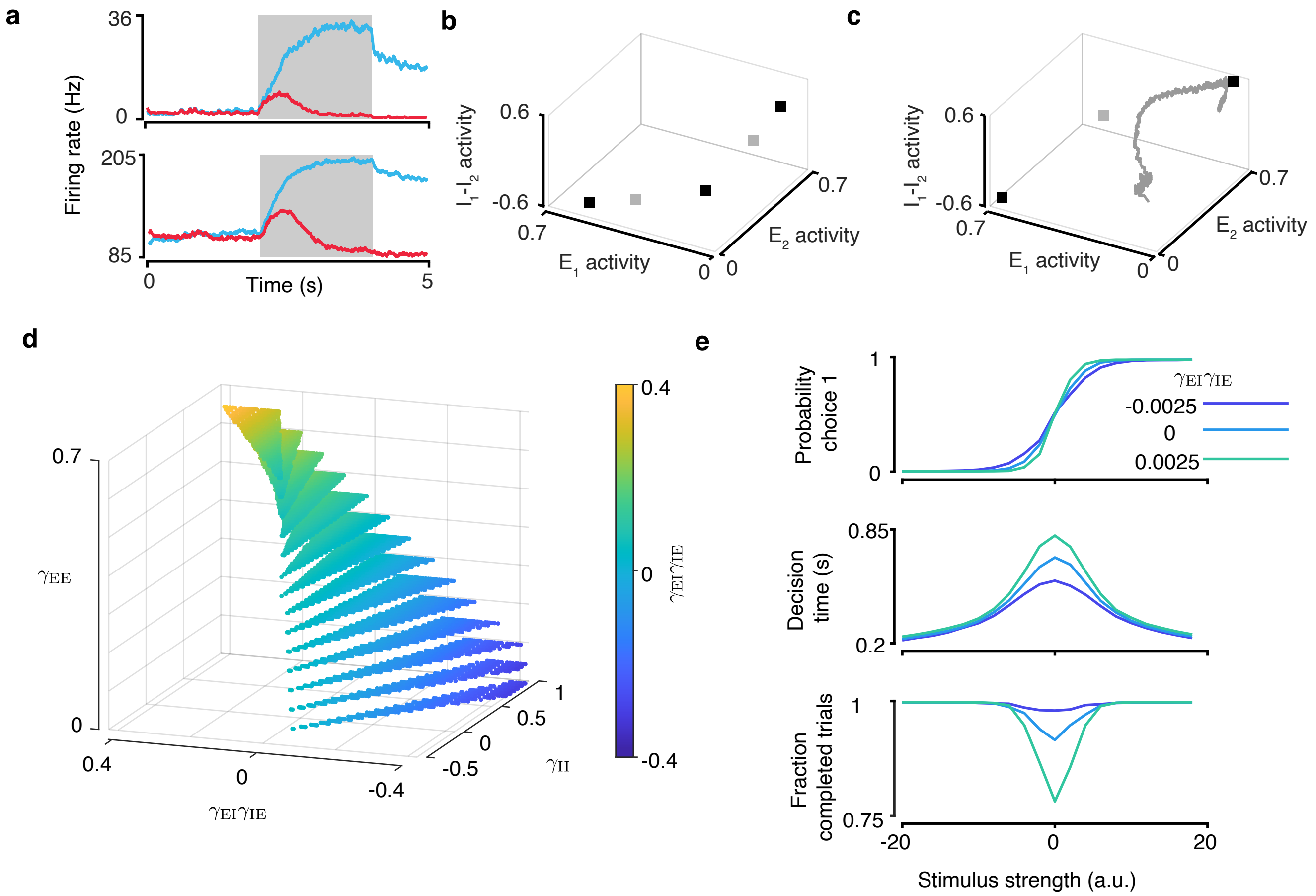


**Supplementary Figure 3. Specificity of inhibitory-to-inhibitory connections and dynamics of GABA synapses have a limited effect on decision-making dynamics.** A four-variable mean-field model with two excitatory and two inhibitory choice-selective populations produces firing rate dynamics similar to the two-variable model. (**a**) Firing rate dynamics for excitatory (upper panel) and inhibitory neurons (lower panel) in a four-variable model are shown for a trial with stimulus strength equal to 0. Specificty parameters are $\gamma_{EE} = 0.32$, $\gamma_{EI} = 0.25$, $\gamma_{IE} = 0$, $\gamma_{II} = 0$. (**b-c**) Similar to the two-variable model, the four-variable model has eight fixed points required for decision-making dynamics: five in the unstimulated phase space (b) and three in the stimulated phase spaces (c). For visualization, the four-dimensional phase space was reduced to three dimensions by taking the difference between activity of two inhibitory populations. Black squares indicate fixed-point attractors, gray squares indicate saddle-points. A trajectory on an example trial is plotted in gray. Same specificity parameters as in a. (**d**) Inhibitory-inhibitory specificity has a weaker effect on the space of circuits supporting decision-making than excitatory-inhibitory specificity. The volume in the specificity parameter space supporting decision-making dynamics is largely defined by the relationship between $\gamma_{EE}$ and $\gamma_{EI}\gamma_{IE}$ with a small impact of $\gamma_{II}$ noticeable as a curl in the volume of parameters supporting decision-making. (**e**) The four-variable model model shows the speed-accuracy trade-off when varying the inhibitory specificity, similar to the two-variable model.

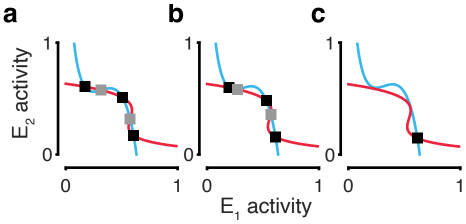


**Supplementary Figure 4. Circuits lacking the saddle point can discriminate easy stimuli.** Phase planes of a strongly ipsispecific circuit ($\gamma_{EE} = 0.3196$, $\gamma_{EI} = 0.25$, $\gamma_{IE} = 0.75$) show that as the stimulus strengths in favor of one of the choices increases, the symmetrical attractor disappears enabling the circuit to make decisions. (**a**) Stimulus strength is 0.0. (**b**) Stimulus strength is 2.5. (**c**) Stimulus strength is 5.0.

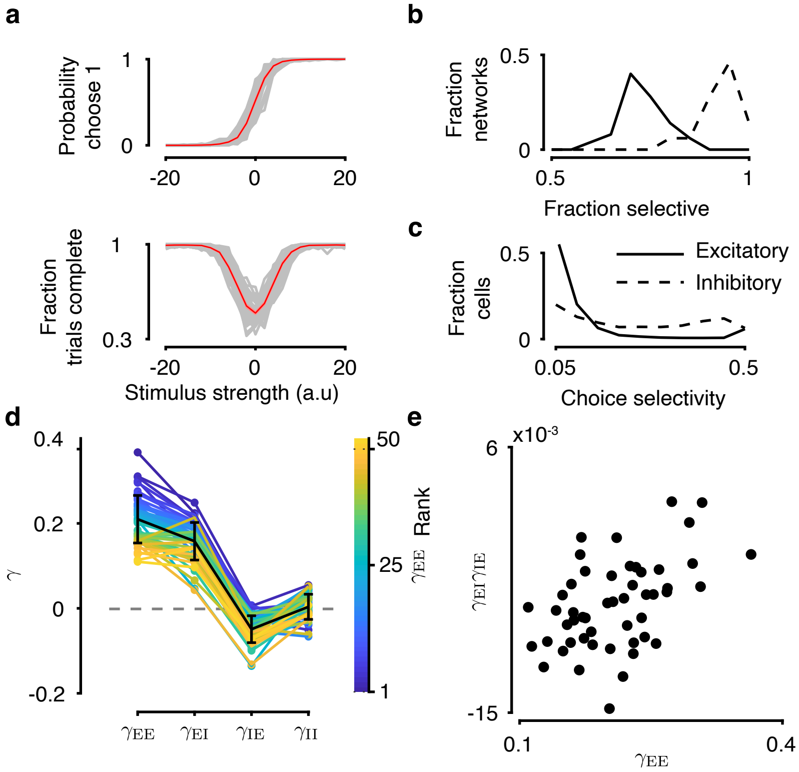


**Supplementary Figure 5. Performance and circuit structure in RNNs with 200 excitatory and 50 inhibitory units.** (**a**) Larger RNNs train to a similar level of performance as smaller networks. Psychometric and chronometric functions for individual trained RNNs (grey) and mean across 50 networks (red). (**b-c**) Trained networks were similar to smaller RNNs in both the fraction of selective units (b) and average choice selectivity (c). Inhibitory units were more likely to be choice-selective than excitatory units. (**d-e**) The resulting circuit structure was similar to smaller RNNs with high $\gamma_{EE}$ and $\gamma_{EI}$ (d, colored lines - individual RNNs sorted by $\gamma_{EE}$, black - mean ± s.d. across 50 networks) and a positive correlation between $\gamma_{EE}$ and $\gamma_{EI}\gamma_{IE}$ (e).

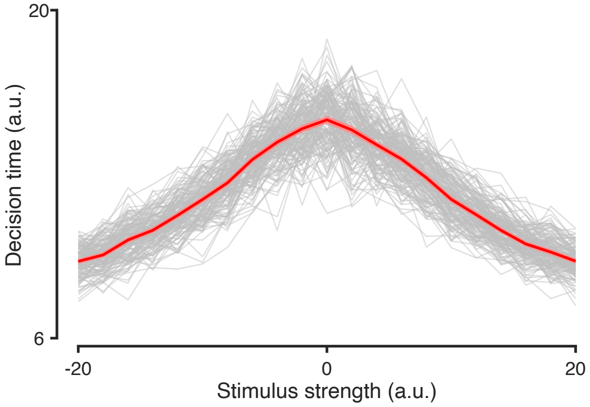


**Supplementary Figure 6. RNNs display a stimulus-strength dependent speed versus accuracy trade-off.** Chronometric functions for individual trained RNNs (grey) and the mean across 150 networks (red). RNNs take longer to report decisions for difficult stimuli (stimulus strength near 0) than for easier stimuli.

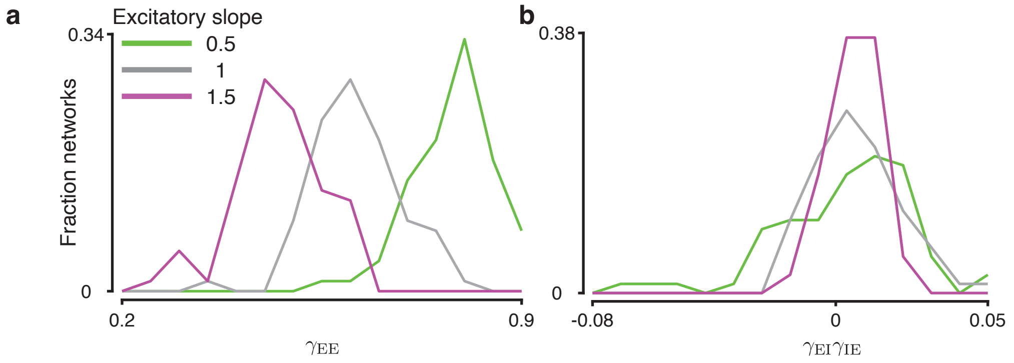


**Supplementary Figure 7. Changes in the excitatory unit excitability result in altered circuit structure in RNNs.** (**a**) Trained RNNs with hypoexcitable excitatory units (green) show higher $\gamma_{EE}$ than RNNs with baseline (gray) or hyperexcitabile units (purple). (**b**) The distribution of the specificity index $\gamma_{EI}\gamma_{IE}$ is similar across all RNNs. In all panels, distributions include 75 networks for each excitability level.

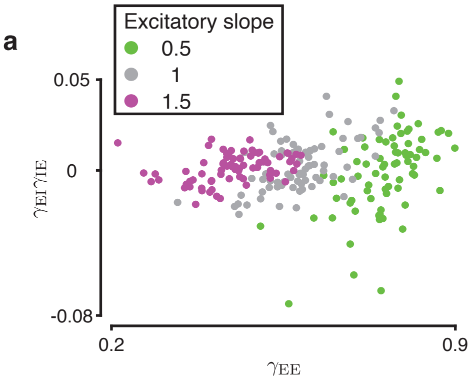


**b**

| Excitatory slope | Pearson's correlation coefficient | p-value |
|---|---|---|
| 0.5 | 0.51 | 2.5e-6 |
| 1 | 0.55 | 3.7e-7 |
| 1.5 | 0.33 | 3.7e-3 |

**Supplementary Figure 8. Excitatory and inhibitory selectivity is correlated in RNNs with different excitability of excitatory units.** $\gamma_{\mathrm{EE}}$ and $\gamma_{\mathrm{EI}}\gamma_{\mathrm{IE}}$ are correlated in RNNs trained with different excitability of excitatory units. (**a**) Hypo- and hyperexcitable networks show a similar relationship between $\gamma_{\mathrm{EE}}$ and $\gamma_{\mathrm{EI}}\gamma_{\mathrm{IE}}$ as the baseline networks. (**b**) For each excitability level, the correlation between $\gamma_{\mathrm{EE}}$ and $\gamma_{\mathrm{EI}}\gamma_{\mathrm{IE}}$ was significant ($p < 0.05$, MatLab corrcoef function, two tailed).

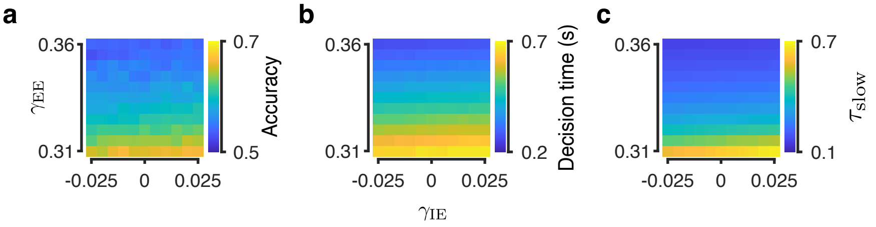


**Supplementary Figure 9. Excitatory selectivity has a larger effect on mean-field circuit dynamics than inhibitory selectivity.** Changes in $\gamma_{\mathrm{EE}}$ (y-axis) have a larger effect on (**a**) accuracy, (**b**) decision time, and (**c**) $\tau_{\mathrm{slow}}$ than inhibitory selectivity (x-axis). Data in a and b represent the mean of 10,000 trials at stimulus strength equal to $-2$. $\tau_{\mathrm{slow}}$ is caluclated at stimulus strength equal to $-2$. $\gamma_{\mathrm{EI}} = 0.25$.

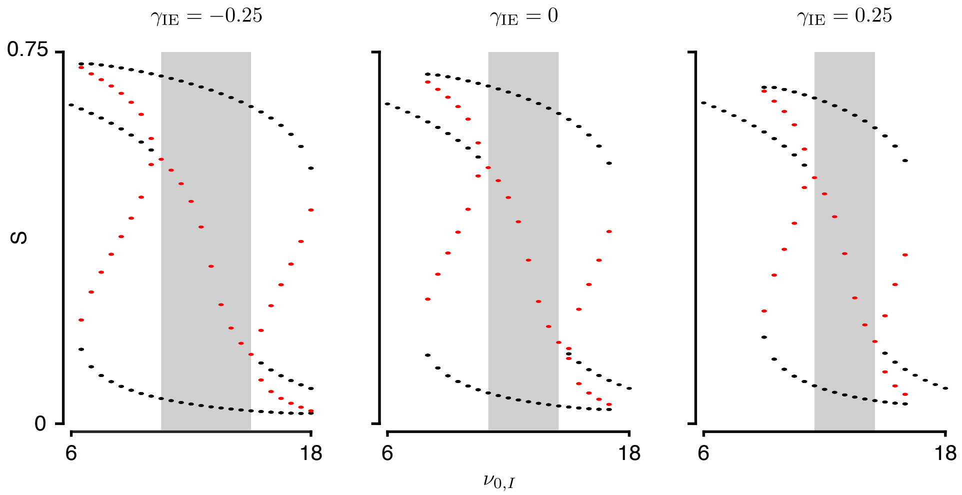


**Supplementary Figure 10. Bifurcation diagrams for circuits with the baseline input to inhibitory neurons as a control parameter.** The location of fixed points for contraspecific (left), nonspecific (center), and ipsispecific (left) circuits. Black points show attractors and red points show saddle points. The grey region indicates the range of the baseline inputs to inhibitory neurons $\nu_{0,I}$ which support decision-making. $\gamma_{\mathrm{EE}} = 0.32$, $\gamma_{\mathrm{EI}} = 0.25$.
